# Cardiovascular Risk in Women With PCOS

**DOI:** 10.5812/ijem.4020

**Published:** 2012-09-30

**Authors:** Pietro Scicchitano, Ilaria Dentamaro, Rosa Carbonara, Gabriella Bulzis, Annamaria Dachille, Paola Caputo, Roberta Riccardi, Manuela Locorotondo, Cosimo Mandurino, Marco Matteo Ciccone

**Affiliations:** 1 Section of Cardiovascular Diseases, Department of Emergency and Organ Transplantation, University of Bari, School of Medicine, Policlinico, Bari, Italy

**Keywords:** PCOS, Cardiovascular Risk, Metabolic Syndrome, Diabetes, Hypertension

## Abstract

Polycystic ovary syndrome (PCOS), or Stein-Leventhal syndrome, is a common endocrine disorder defined by two of the three following features: i) oligoovulation or anovulation, ii) clinical and/or biochemical signs of hyperandrogenism, or iii) polycystic ovaries, once the related endocrinological and gynaecological disorders have been excluded. PCOS does not exclusively involve the reproductive apparatus , it has a complex number of systemic relevancy symptoms. It leads to Metabolic Syndrome, with severe consequences on the cardiovascular apparatus. Many clinical studies have underlined the connection between PCOS and the cardiovascular risk profile of such female patients, due to a lipid/glucose altered metabolism, hypertension, systemic inflammatory condition (assessable by markers such as VES, TNF-alfa, citokines and C-reactive protein (hsPCR) levels), and vascular injuries. Considering the early onset of the disease, PCOS could be considered as a real cardiovascular risk factor which affects the quality of life seriously. The current review aimed to point out the main connections between PCOS and cardiovascular risk factors according to the latest findings coming from literature data analysis, and try to depict the great influences that such a common disease can have on the patients’ health integrity.

## 1. Introduction

Polycystic ovary syndrome (PCOS), or Stein-Leventhal syndrome, is a common endocrine disorder that affects 5-10% of reproductive-age women (1), and according to 2003 Rotterdam criteria (2), it is commonly defined by two of the following three features: i) oligoovulation- or anovulation, ii) clinical and/or biochemical signs of hyperandrogenism, or iii) polycystic ovaries, once related endocrinological and gynaecological disorders have been excluded.

The current review aimed to analyze the actual knowledge of how the metabolic disorder, a PCOS feature, affects cardiovascular risk factors in women suffering from this disease.

## 2. Physiopathology

The pathophysiological mechanisms underlying the syndrome are apparently determined by the complex interaction between the functionality of the hypothalamic-pituitary-ovarian or hypothalamic-pituitary-adrenal axis and metabolic disorders, such as obesity, insulin resistance and compensatory hyperinsulinemia (3). These factors are associated with a genetic predisposition, confirmed by the identification of abnormal gene clusters involved in steroidogenesis and regulation of peripheral insulin sensitivity (4, 5). Various pathogenic hypotheses try to explain the increased peripheral availability of androgen hormones, which depend on excessive ovarian and adrenal production (6). The conversion of androgens into estrogens results in an estrogenic acyclic pattern that alters the pulsatile secretion of gonadotropins. This altered LH / FSH ratio determines the typical ovarian thecal hyperplasia with a further increase in androgens and the subsequent vicious cycle (7). The hyperinsulinemia per se is considered as a possible cause of an increased ovarian androgen production, which alters gonadal steroidogenesis process ,both directly and indirectly (8). Moreover, in overweight and obese women, the presence and activity of aromatase, a conversion enzyme located in fat cells, causes an excessive peripheral aromatization of androstenedione with consequent iperestronemia, capable of altering the pulsatile release of gonadotropins (9). Obesity is also associated with insulin resistance and compensatory hyperinsulinemia, maintaining the vicious circle.

## 3. PCO, Lipids and Glucose Dysmetabolism

Insulin resistance (IR) is common in both obese and lean PCOS women (70%), with a 7.5-10% predominance of type 2 diabetes mellitus (DM) against 0.7% in non-PCOS young healthy women with type 2 DM. The conversion rate from impaired glucose tolerance to frank diabetes is 5 to 10fold higher in women with PCOS compared with non-PCOS women both in the United States and Australia (10, 11).

The role of IR and insulin secretion in the pathogenesis of glucose intolerance in PCOS was studied in 11 adolescents with IGT and 10 with normal glucose tolerance (NGT) by Arslanian et al. (12). Great abnormalities both in insulin action and in insulin secretion can be observed in obese adolescents with PCOS. Glucose intolerance is associated with 1) decreased first phase insulin secretion, 2) decreased glucose disposition index, and 3) increased hepatic glucose production. These metabolic abnormalities are precursors of type 2 DM (as manifested by the significant impairment of the β-cell function) which compensates for severe peripheral IR and elevated hepatic glucose production in women with impaired glucose tolerance (IGT).

Disturbances in insulin action are particularly related to PCOS pathogenesis. Ming Li et al. (13) studied insulin receptors in the skin fibroblasts of PCOS patients. They indicated a possible mechanism involved in IR: the decreased insulin-dependent receptor, tyrosine phosphorylation, and the increased constitutive receptor, serine phosphorilylation, probably associated with a cell membrane secondary factor, which can inhibit insulin receptor signaling. This inhibition is responsible for glucose transporter-4 reduced transfer to the cell membrane and, consequently, glucose intake reduction.

Normally, the binding of insulin to the insulin receptor induces the beta subunit tyrosine kinase activation. Thus, the insulin receptor substrates-1/-2 (IRS-1/IRS-2) are phosphorylated, and the phosphatidylinositol-3-Kinase is activated. The final step is glucose transporters translocation to the cell membrane and the glucose uptake stimulation (13).

The protein kinase A can probably induce the serine phosphorylation of the receptor by creating a serine phosphorilylation of 17-alpha hydroxylase, which is an important enzyme in theca cells androgenesis. This can partially explain hyperandrogenism pathogenesis in PCOS.

Furthermore, insulin stimulates thecal cells which in turn stimulate testosterone biosynthesis in women with PCOS by activating receptors and using inositol-glycan mediators as the signal transduction system (14).

Hyperglycaemia is linked to systemic inflammation as observed in glucose-intolerant PCOS women. Hyperglycaemia and insulin resistance could effectively induce an increase in reactive oxygen species (ROS) concentration (15). ROS activate the nuclear factor kappa-light-chain-enhancer of activated B cells (NFkB), which are able to stimulate inflammatory factor synthesis (i.e. Tumor Necrosis Factor-α [TNF-α]),that is a mediator of insulin resistance, and is highly expressed in PCOS patients. TNF-α production is independent of obesity, although closely related to hyperglycemia: Gonzalez et al. have indicated that the increased activation of NFkB triggers stimulate inflammation and IR, and consequently hyperandrogenism in PCOS (16).

IR appears to contribute to hyperandrogenism and other gonadotropin abnormalities through two mechanisms: 1)a hyperinsulinemic state reduces circulating sex hormone binding globulin (SHBG) levels, causing an increase in bioavailable (free) testosterone since less SHBG is available to bind with testosterone; 2) high insulin concentrations stimulate androgen biosynthesis through ovaries (14, 17): insulin decreases Insulin Growth Factor binding globulin 1 (IGFBP-1) and increases free IGF and thus increases ovarian theca cell proliferation and androgenesis as well.

Moreover high levels of insulin can reduce the Aromatase’s activity. High levels of estrogen and low levels of testosterone are indicating high levels of aromatase activity. Glucose intolerance and IR lead to low dehydroepiandrosterone (DHEA) , and high insulin levels, thus, testosterone increase probably causes the typical symptoms of PCOS: acne, hirsutism and masculinisation (18).

Therefore the presence of IR, and cardiovascular risk, may be determined by adopting variables associated with insulin action. The Lipid Accumulation Product (LAP) index could be useful in this sense (19). The LAP index is a value that combines two parameters: waist circumference (WC), i.e. the measurement of trunk fat which includes the intra-abdominal deposit, and fasting triglycerides concentration .

Its determination is given by the following formula (19):

[WC (cm) – 58] x triglycerides (mmol/l)

According to Wiltgen, LAP identifies the presence of IR when it is > 34.5 (sensitivity: 84%; specificity 79%) (20).

Furthermore, high triglyceride level is linked to an increased homeostasis model assessment (HOMA) index (calculated as fasting insulin concentration × fasting plasma glucose concentration/22.5) at any age, even in young women. This risk marker has a strong association with each criteria defining metabolic syndrome and the LAP index is highly correlated with the HOMA index in patients with PCOS (21). PCOS patients have higher values of LAP index than controls with the same BMI, they are insulin resistant and have a higher metabolic syndrome prevalence (20). The LAP index is also associated with IGT (22). In fact, an Austrian study, demonstrated that in PCOS women, age, BMI, blood pressure, fasting and post prandial glucose insulin and free testosterone tend to increase with the LAP index growth, while SHBG decreases. The cut-off value to predict IGT presence was 44.1 (22).

Finally, literature dealt with IR in women with PCOS and metformin therapy treatment, but the conclusionswere controversial (23, 24). Literature data (25) on the effects of simvastatin on the insulin sensitivity and the fasting insulin are not conclusive. The difference in the response to statin therapy in PCOS could be due to a transient phenomenon or the characteristics of the studied population (24).

Therefore, the treatment of these young women, should require dietetic interventions. A diet based on the consumption of carbohydrates with a low glycemic index (LGI) has positive effects on cardiovascular risk reduction (26).

McMillan-Price et al. (26) considered several dietetic regimens which were different in their protein content and glycemic index. The results indicate that, even if there was a loss in body weight in all the diets, the reduction of cardiovascular risk occurred only in patients treated by an LGI diet, as indicated by lowering the Low Density Lipoprotein (LDL) cholesterol (26).

Marsh et al. (27) recruited PCOS participants following either a low-fat, LGI diet (on the basis of LGI breads and cereals) or a low-fat, conventional healthy diet on the basis of high-fiber and moderate- to-high glucose index breads and cereal foods. A significant improvement of insulin sensitivity was observed only in the LGI group (the insulin sensitivity index [ISI]OGTT was 2.2 ± 0.7, con P < 0.01 compared with baseline values). Furthermore, women who took metformin with the LGI diet showed a greater increase in insulin sensitivity than those consuming a conventional diet alone (4.3 ± 1.0 vs 1.1 ± 0.7, P = 0.12). Additionally, the improvement of ISIOGTT did not have the same force in subjects with conventional healthy diets and metformin, in comparison to subjects with conventional healthy diets alone (0.2 ± 1.1 vs 1.0 ± 1.0. P = 0.62) (21).

Therefore, an LGI diet brings about greater beneficial effects than the one with an elevated glycemic index. This diet probably develops a low postprandial glycemia and insulinemia. Further studies are necessary.

## 4. PCOS and Hypertension

According to the World Health Organization definition, arterial hypertension (AH) is defined as systolic (SBP) and (DBP) diastolic blood pressure > 140 and > 90 mmHg respectively and/or use of anti-hypertensive drugs. (28). A study conducted in North East Brazil (29) on women with and without PCOS, indicated an association between AH and this endocrine disease, revealing a 2-fold prevalence of AH in the group with PCOS (18.6 vs. 9.9, P < 0.05). IR and hyperinsulinemia can explain AH pathogenesis in PCOS: they alter vascular smooth muscular cells causing hypertrophy of vascular smooth muscle with reduced compliance, the interference in the endothelium-dependent vasodilatation mechanism, the activation of the renin-angiotensin-aldosterone system and sodium retention.

Chen and Yang (30) found that high testosterone levels (free androgen index: > 19 %) and no SHBG, increased the risk of elevated SBP and DBP values (Odds ratio [OR]: 3.817, P = 0.029; 95%, Confidential Interval [CI]: 1.14 to 12.74) even when adjusted for age, body mass index, and other anthropometric, metabolic and hormonal variables.

Studies conducted by Lecke SB, Morsch DM and Spritzer PM in Hospital de clinicas de Porto Alegre (HCPA) demonstrated a link between CYP19 gene expression, levels of aromatase and blood pressure (31): androgen excess may be involved in the high levels of CYP19, a gene encoding for the enzyme aromatase, expressed in abdominal tissue fat. A high expression of this gene, induces low estrogen and high androgen concentrations. Furthermore, subcutaneous CYP19 mRNA was higher in hypertensive PCOS than in control and normotensive PCOS women (P < 0.014). The CYP19 gene expression correlated positively with SBP (P = 0.006) and DBP (P = 0.009) ]

A transversal study of the Tunisian National Institute of Nutrition demonstrated that a family history of hypertension was surely not a predictive factor for the same disease in PCOS patients; women with PCOS and hypertension had a significantly higher BMI and triglyceride levels than those with PCOS but without hypertension; whereas they did not differ in their androgens and total cholesterol levels (32).

Even considering PCOS patients without a frank hypertension, a Swedish study showed that PCOS women had higher day-time SBP (P < 0.05), mean arterial values of blood pressure (P < 0.06) and an increased pulse rate (P < 0.05) than controls. Nevertheless, the groups did not differ in DBP (P = 0.05). Moreover women with PCOS had high values of day-time blood pressure also after adjusting for BMI, body fat distribution and insulin resistance, so these results may suggest a blood pressure liability that may indicate a pre-hypertensive state (33).

An interesting association between PCOS and a non–dipping blood pressure pattern in young women has been underlined by Kargili: patients are defined dippers if their nocturnal SBP falls by 10 % or more compared to the day-time SBP, likewise when SBP falls by < 10 % compared to daytime SBP, they are defined non –dippers (34).

A non dipping pattern is prevalent in patients with PCOS even if they are young and non obese (43.4% in PCOS patients and 3.9 % in the control group); probably this pattern, like the autonomic dysfunction and IR, may be associated with endothelial impairment, oxidative stress, left ventricular hypertrophy and renal dysfunction (35).

Moreover, although HOMA-IR values and waist circumference are strongly connected with non dipping pattern and PCOS, the lipid profile and BMI are not; so the study revealed that a non dipping pattern is prevalent in PCOS patients even if they were young and non obese; (34).

Drugs for hypertension treatment in PCOS have been considered. Agarwal et al. administrated metformin to a group of young women with PCOS, randomly receiving the drug or the placebo. After 12 weeks of treatment, 8 weeks of washout, and finally the reversal of the treatments in the two groups, arterial pressure in metformin groups reduced to normal values, both in peripheral systolic and diastolic pressure and central systolic and diastolic pressure values, which demonstrated a close relationship between insulin resistance and a hypertensive condition in PCOS patients (23).

## 5. PCOS and C - Reactive Protein

Serum levels of highly sensitive C-reactive protein (hsCRP), a vascular inflammatory marker, may predict the development of CVD (36) and type 2 diabetes (37).

Some studies report that hsCRP levels are higher in women with PCOS, confirming the hypothesis that diabetes induced CVD by chronic inflammation in PCOS patients (38).

In contrast, Mohlig et al. (39) reported that serum hsCRP levels are associated with obesity rather than with the presence of PCOS per se. Thus, the serum hsCRP levels were increasing in women with PCOS and the variables predicting serum hsCRP levels remained unclear. Furthermore hsCRP levels were positively correlated with waist circumference (r = 0.46, P < 0.01), BMI (r = 0.46, P < 0.01), SBP (r = 0.42, P < 0.05) and DBP (r = 0.39, P < 0.05), and tended to be negatively correlated with insulin-mediated glucose uptake (r = -0.31, P = 0.07) in women with PCOS.

Ji Young Oh compared hsCRP levels in lean women with or without PCOS in order to determine whether hsCRP was associated with CVD risk alone or it was related to fat distribution. The study suggested that PCOS was not associated with increased CRP levels. Only normal-weight women with PCOS who had been recruited to minimize the effects of obesity on hsCRP levels A multiple regression analysis showed that BMI, systolic blood pressure, and insulin-mediated glucose uptake predicted a higher level of hsCRP (38).

As previously reported, obesity appeared to be a major factor associated with elevated hsCRP in individuals with metabolic syndrome (40). Moreover, visceral fat correlated with hsCRP concentrations independent of total adiposity (41).

Since insulin resistance was frequently associated with PCOS, insulin-sensitizing interventions might theoretically have beneficial effects on hsCRP levels. Rosiglitazone seemed to reduce high-sensitivity hsCRP levels and endothelial dysfunction in PCOS patients (42).

In fact, the drug induced a significant improvement in endothelium-dependent (flow-mediated vasodilatation [FMD]) vascular responses after the rosiglitazone treatment (9.9 + 3.9 vs 16.4 + 5.1%; P < 0.01). The baseline artery diameter and endothelium independent vascular responses did not change with the treatment. An endothelium dependent vasodilatation which was significantly correlated with insulin resistance and hsCRP levels (P = 0.05) (43). Haffner et al. (44) showed that rosiglitazone reduced the serum levels of the pro-inflammatory marker hsCRP in patients with type 2 diabetes. These data indicate that a reduction in insulin resistance may have a cardioprotective effect, even in non-obese women with PCOS.

Furthermore, metformin was not able to reduce the inflammation state in PCOS women (23), although Banaszewska et al., by comparing the administration of simvastatin and metformin in such patients demonstrated an improvement in the condition of systemic inflammation: there was a reduction in hsCRP levels in comparison to the plasmatic amounts presented at the beginning, significant in the group with simvastatin (-34%; P < 0.01), in the subjects treated with metformin therapy alone (-52%; P < 0.01) and in women who took both (-47%, con P = 0.01) (24).

Further studies are needed in order to reach a full definition of the PCOS aspect.

## 6. PCOS and Homocysteine

An increase in plasma homocysteine (Hcy) is a deleterious metabolic effect of hyperinsulinemia in the general population. As it was just indicated, hyperinsulinemia and insulin resistance are some of the main characteristics of women with PCOS (22, 27). Plasma levels of insulin seem to influence Hcy metabolism by affecting glomerular filtration or important enzymes activity in Hcy metabolism [i.e., Methyltetrahydrofolate Reductase (MTHFR) and hepatic Cystathione β-Synthase (CBS)] (45, 46). Plasma Hcy levels are widely accepted as an independent risk factor for cardiovascular disease (47). Yarali et al. (48) pointed out the cardiac diastolic dysfunction in PCOS patients as detected by echocardiography, and indicated a significantly higher plasma Hcy level in both lean and obese PCOS women, and it was related to insulin resistance. Schachter et al. (49) also reported that insulin resistance and hyperinsulinemia in patients with PCOS were associated with elevated plasma Hcy levels regardless of body weight. Furthermore, elevated plasma Hcy levels in PCOS are independent of BMI (50).

Elevated Hcy levels induce the injury of endothelial cells, the proliferation of muscle cells, an increased inflammatory cytokine expression/activity and atherogenesis; moreover, they induce the vulnerability of established atherosclerotic plaque (51).

Hcy levels in women with PCOS are reduced after 3 months of folic acid therapy (52).

The administration of insulin sensitizers, such as metformin and rosiglitazone, which were proposed in PCOS patients to improve ovulation induction and metabolic parameters, increased serum Hcy levels in patients with type 2 diabetes mellitus, despite the decrease in insulin resistance (53). Hcy levels increased in PCOS patients treated with metformin, irrespective of smoking and obesity (54). The treatment with rosiglitazone increased Hcy levels, as well. This effect may be explained by folate depletion (55) and, therefore, may be prevented by a concomitant folate supplementation (56, 57). As a result, folate supplementation may reduce cardiovascular risks in these patients.

Statins seems to decrease serum Hcy levels in PCOS patients (58). Many studies have demonstrated that oral contraceptives containing 35 μg ethynylestradiol and 2 mg cyproterone acetate lead to a fast decrease in Hcy levels in non-smoking PCOS women (59-61). In some of these trials, Hcy concentrations increased in the smokers, whereas they decreased in non-smokers. However, the influence of smoking on Hcy was not statistically significant . Oral contraceptives containing drospirenone did not affect Hcy levels in patients with PCOS during the 6 month therapy (62).

The available data so far indicate that coronary heart diseases, as well as cerebrovascular diseases, are more common in postmenopausal PCOS patients. Persisting high androgen levels also through the menopause, obesity and onset diabetes mellitus are the main mechanisms accounting for this risk (63). After menopause, basal Hcy levels increase progressively (64) and this could partially explain the cardiovascular risk burden of such patients, although hormone replacement therapy reduces Hcy concentrations (65).

ADMA (Asymmetric Dimethylarginine) is considered as an endothelial dysfunction marker (66). Its plasma concentration was significantly higher in women with PCOS than in healthy control groups (67). The possible mechanism of elevation of ADMA in hyperhomocysteinemia includes a reduced renal excretion (68) and a decreased activity of the hydrolase enzyme, which metabolizes ADMA (69). Therefore, there is a correlation between Nitric Oxide (NO) bioavailability, hyperhomocysteinemia, chronic inflammation, renal insufficiency and hyperandrogenemia in the pathogenesis of the cardiovascular disease responsible for the increased cardiovascular risk in women with PCOS.

## 7. PCOS and Vascular Injury

PCOS is able to impair vascular integrity. Ciccone et al. (70) demonstrated an increase of the antero-posterior diameter infrarenal abdominal aorta (APAO) in PCOS patients (13.87 ± 1.8 12.18 ± 2.3 PCOS against a control with P < 0.01) considering the young age of the study group (age: 17-27 years). It is the largest and earliest damage of the wall, that precedes the appearance of the alterations on other vessels, such as the wall thickening of the carotid (71, 72) and femoral arteries, the changes of which were not statistically significant.

Another expression of vessels injury is central arterial stiffness (73, 74). It is characterized by an excessive collagen in the vessel wall, an amount higher than the presence of elastin, induced by the inflammatory state and the increase in luminal pressure. A key role is also played by metal-proteinases that degrade the matrix proteins (28). Furthermore, the glycation phenomenon makes collagen more rigid, decreases elastin and stimulates inflammation (75, 76).

A strong correlation was found between increased arterial stiffness (measured by non-invasive methods such as the augmentation index and PWV, that is Pulsed Wave Velocity) in conditions of obesity and PCOS: in the experience of Meyer et al. (77) the arterial stiffness and endothelial dysfunction increased in overweight subjects with PCOS (PWV, 7.4 + 0.1 vs. 6.6 + 0.2 m/sec) and endothelial dysfunction (FMD, 9.8 + 0.4 vs. 13.3 + 0.9), compared with controls.

Dessapt Baradez-C et al. (78) demonstrated an increased arterial stiffness in PCOS women with BMI < 30, with a significant increase of the augmentation index (18.4 ± 1.9% vs. 4.9 ± 2.0%) also attributed to a reduction in the vascular progenitor CD34 (+) 133 (+) VPCs (317.5 ± 51.0 vs. 558.3 ± 101.2, P = 0.03) (79, 80).

On the other hand there are numerous studies that have reached opposite conclusions, emphasizing the presence, in normal-weight PCOS women, of endothelial dysfunction (81, 82).

Metformin positively influences endothelial dysfunction, improving arterial stiffness (23). It does not work by lowering levels of IR and androgen hormones (no significant data for HOMA-IR, Testosterone, Free Androgen Index and SHBG). It is suggested that metformin can modulate the endothelial dysfunction neither improving the insulin sensitivity, nor reducing androgen hormones effects on the endothelium, but probably through the activation of an adenosine monophosphate kinase in the endothelial cells and in the vascular smooth muscle (23).

The administration of metformin is capable of inducing a significant improvement in arterial stiffness in these women (23).

Banaszewska et al. compared the administration of simvastatin and metformin in women with PCOS, and observed endothelial dysfunction improvement by reducing Soluble Vascular Cell Adhesion Molecule (8.3%, P < 0.01, with simvastatin; 6.6%, P = 0.02, with metformin; 4.5%, P = 0.03, with both simvastatin and metformin) (24).

## 8. Conclusions

PCOS patients clearly present a higher risk of cardiovascular diseases, linked to metabolic dysfunction due to its peculiar hormonal pattern, characterized by hyperandrogenism, insuline resistance, dyslipidemia, and inflammatory state. Apart from well-known cardiovascular risk factors (hypertension, diabetes, inflammation and hypercholesterolemia), the recent aforementioned studies underline interesting common features among these patients. PCOS women appear to have a “non dipping” pattern of blood pressure (even without hypertension) and increased serum levels of homocysteine. Moreover, even if the role of PCOS in arterial stiffness is controversial, the antero-posterior diameter of the abdominal aorta looks like a useful means to assess risks in these patients.

An aspect that could be deepened is the possible role of dietetic and drug treatments of metabolic disorders in improving the cardiovascular risk in PCOS woman. Therefore further study is needed in order to improve the knowledge on such a subject.
